# Risk factors for chronic hypertension 5 years after a pregnancy complicated by preeclampsia: a systematic review and meta-analysis

**DOI:** 10.1097/HJH.0000000000003995

**Published:** 2025-02-27

**Authors:** Lotte W. Voskamp, Melek Rousian, Joni J. Koerts, Régine P.M. Steegers-Theunissen, A.H. Jan Danser, Koen Verdonk

**Affiliations:** a Department of Obstetrics & Gynecology; b Division of Vascular Medicine and Pharmacology, Department of Internal Medicine, Erasmus MC, University Medical Center Rotterdam, The Netherlands

**Keywords:** chronic hypertension, hypertensive disorders of pregnancy, postpartum, preeclampsia, risk factors

## Abstract

Approximately 30% of women with a history of preeclampsia develop chronic hypertension within 10 years of pregnancy. This systematic review summarizes risk factors before, during, and immediately after pregnancy for the development of chronic hypertension 5 years after preeclampsia. Databases were searched with terms ‘preeclampsia’ and ‘postpartum hypertension’ or ‘cardiovascular disease’ up to 30th October 2023. Observational studies reporting chronic hypertension more than 5 years after preeclampsia were included. Quality was assessed using the Newcastle–Ottawa scale. Wherever possible, a meta-analysis was conducted. Twenty-one cohort and five case–control studies, with a median quality score of 8/10, were included, involving 197 793 patients and reporting 32 risk factors. Preeclampsia in a subsequent pregnancy is associated with chronic hypertension [risk ratio (RR) 2.26, 95% confidence interval (CI) 1.59–3.22, *n* = 45 626]. Other significant risk factors include early-onset of preeclampsia (<34 weeks gestation), maternal BMI, blood pressure, diabetes, and family history of hypertension.

## INTRODUCTION

The incidence of preeclampsia has increased over the past decade, and it is now widely acknowledged that the morbidity and mortality associated with preeclampsia are not limited to pregnancy [1]. Women with a history of preeclampsia are at an increased risk of developing cardiovascular disease later in life, possibly mediated by chronic hypertension, the most important modifiable risk factor for cardiovascular disease [1]. Approximately 30% of women with preeclampsia develop chronic hypertension within 10 years of pregnancy, which is six times higher than that observed in normotensive pregnancies [2,3]. However, how to identify women at the highest risk of these long-term complications and who would benefit most from postpreeclampsia surveillance remains unknown.

It is postulated that acute vascular damage and atherosclerosis, triggered by endothelial dysfunction, inflammation, and oxidative stress in preeclampsia, contribute to the development of chronic hypertension [1,4]. Additionally, the link between preeclampsia and chronic hypertension could be attributed to genetic predisposition and shared risk factors, such as obesity, diabetes, and renal disease [1,4,5]. This association is further emphasized by the increased risk of preeclampsia in individuals with prepregnancy chronic hypertension.

To reduce the impact of preeclampsia on cardiovascular health later in life, organizations such as the American Heart Association and the Lancet Women and Cardiovascular Disease Commission have stressed the significance of enhancing postpregnancy surveillance to prevent cardiovascular events [6,7]. However, standardization of surveillance programs, follow-up frequency and topics covered during visits, remains a subject of debate. A tailored approach to postpregnancy care, rather than a one-size-fits-all approach, may be preferable, especially in the context of healthcare costs and the burden on patients from unnecessary follow-up visits. Ideally, early postpartum risk assessment can offer insights into chronic hypertension risk, educate patients on health and lifestyle, and help tailor follow-up care for those at higher risk. To achieve this goal, it is essential to acquire knowledge of all (non)modifiable risk factors that contribute to chronic hypertension after pregnancies complicated by preeclampsia. Previous systematic reviews have largely focused on determining the short-term incidence of chronic hypertension following preeclampsia. Additionally, two reviews reported one risk factor for the development of chronic hypertension, namely, experiencing preeclampsia in more than one pregnancy compared to only in the first pregnancy [1,8]. However, to the best of our knowledge, no reviews are currently available on risk factors for chronic hypertension as primary outcome. Several studies have demonstrated that the cumulative incidence and hazard ratios for chronic hypertension increase over the first decade following preeclampsia [2,9,10]. Consequently, to capture this prolonged period of increased risk, we extended our follow-up period to at least 5 years after birth for a more comprehensive assessment. Hence, this systematic review aimed to provide a comprehensive summary of factors associated with chronic hypertension at least 5 years after pregnancies complicated by preeclampsia, with the aim of guiding decisions on which women require postpregnancy surveillance.

## METHODS

### Design

This systematic review was conducted at the Erasmus MC, University Medical Center, Rotterdam, the Netherlands, according to the guidelines outlined in the Meta-analysis of Observational Studies in Epidemiology (MOOSE) [11]. The literature search was performed on 30 October 2023, and included the following databases: Embase, Medline (Ovid), Cochrane Central, Web-of-Science, and Google Scholar. The search terms used were ‘preeclampsia’ and ‘postpartum hypertension’ or ‘cardiovascular risk’ (Appendix 1). This review was restricted to studies published in English, with no limitations on the date of publication. The protocol for this review was registered in the PROSPERO International Prospective Register of Systematic Reviews with ID CRD42023412134.

This research focused on observational studies, including cohort and case–control designs, involving women with preeclampsia. Preeclampsia was defined as the presence of new-onset hypertension (SBP ≥140 mmHg and/or DBP ≥90 mmHg) after 20 weeks of gestation and proteinuria (24 h urine protein excretion exceeding 300 mg or a spot protein-to-creatinine ratio greater than 0.3 mg/mg), fetal growth restriction or maternal end-organ dysfunction [12]. The primary outcome was the incidence of hypertension 5 years postpartum, defined as SBP at least 140 mmHg and/or DBP at least 90 mmHg, or the use of antihypertensive drugs [13]. As the definitions of preeclampsia and hypertension can differ between countries and over time, these were compared between the included studies.

Studies were excluded if they lacked sufficient outcome data, were nonpeer-reviewed, were found to have a high risk of bias following assessment, or included women with diagnosed preexisting hypertension (SBP ≥140 mmHg and/or DBP ≥90 mmHg, or use of antihypertensive drugs) and did not adjust for this in the analyses. We did not exclude articles that included multiple pregnancies to avoid limiting the available literature.

We sought to identify the potential risk factors associated with the development of hypertension after preeclampsia. Therefore, pregnancy-related clinical data were collected, including maternal characteristics, such as age, BMI, ethnicity, and smoking status, as well as relevant obstetric and medical history. Descriptive statistics and effect measurements, that is, hazard ratios, odds ratios, and RR, were collected from the included studies. Whenever possible, risk ratios (RR) were calculated to gain insight into the relative risks associated with specific exposures. When crude patient data were not available in the included studies, we attempted to contact the authors via email.

Studies were identified and screened according to the Preferred Reporting Items for Systematic Reviews (PRISMA) 2020 statement for systematic reviews [14]. The initial screening of the title and abstract was performed independently by two researchers (L.W.V. and J.J.K.). Both researchers independently extracted data, and their extractions were compared to identify inconsistencies. Any discrepancies in full-text screening or data extraction were resolved through discussion, with involvement of a third author (M.R.). The extracted data included authors, year, country, number of patients included, population characteristics (e.g. women with preeclampsia or subgroups), study aim, type and methodology (e.g. cohort or case–control), exclusion criteria, confounders, patient characteristics, clinical outcomes (e.g. hypertension risk, cardiovascular/metabolic complications), and blood pressure measurements. We used the Covidence systematic review software Veritas Health Innovation (Melbourne, Australia) for full-text screening and data extraction.

### Quality assessment

The quality of the included studies was evaluated using the Newcastle–Ottawa Scale (NOS) for assessing the quality of nonrandomized studies in meta-analyses [15]. This tool assesses cohort or case–control study quality in three areas: group selection, group comparability, and ascertainment of exposure or outcome. Studies could earn up to nine stars, with 7–9 stars rated as Good Quality, 4–6 as Fair Quality, and 0–3 as Poor Quality. Two reviewers (L.W.V. and J.J.K.) conducted the assessments, and any disagreements were resolved with a third author (M.R.) until consensus was reached.

### Statistical analysis

Risk and hazard ratios are presented in the original article. If no summary effect measures were reported, the risk ratios were synthesized using the crude numbers reported in the article. A meta-analysis was performed using a random-effects model if exposure was described in at least three individual studies, and suitable data were available. When multiple studies described patients from the same study population, we included only the largest study in the meta-analysis. For narrative synthesis, we noted all relevant studies, including those with overlapping populations, to provide a comprehensive overview of the risk factors. *I*^2^ was calculated to assess the heterogeneity. Publication bias was evaluated by visually inspecting funnel plots for asymmetry. Analyses were conducted using R version 4.3.0, and the package ‘meta’ version 6.5-0.

## RESULTS

### Study selection process

**FIGURE 1:**
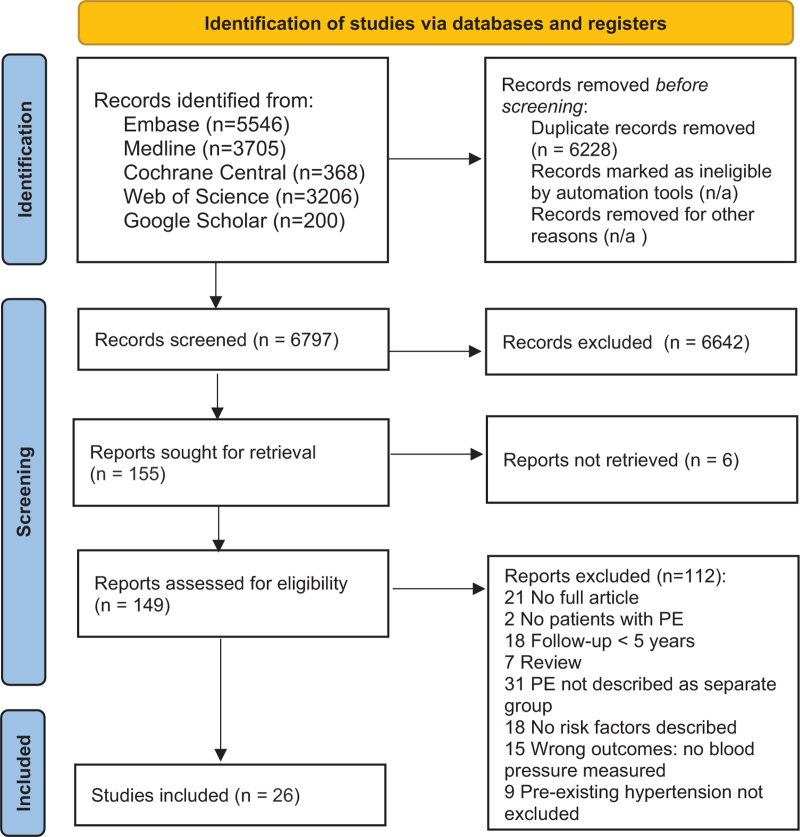
PRISMA flow chart.

Figure 1 presents the PRISMA flow diagram illustrating the initial identification of studies, screening process, and reasons for exclusion. Finally, 26 studies were included in this systematic review.

### Characteristics of included studies

**FIGURE 2:**
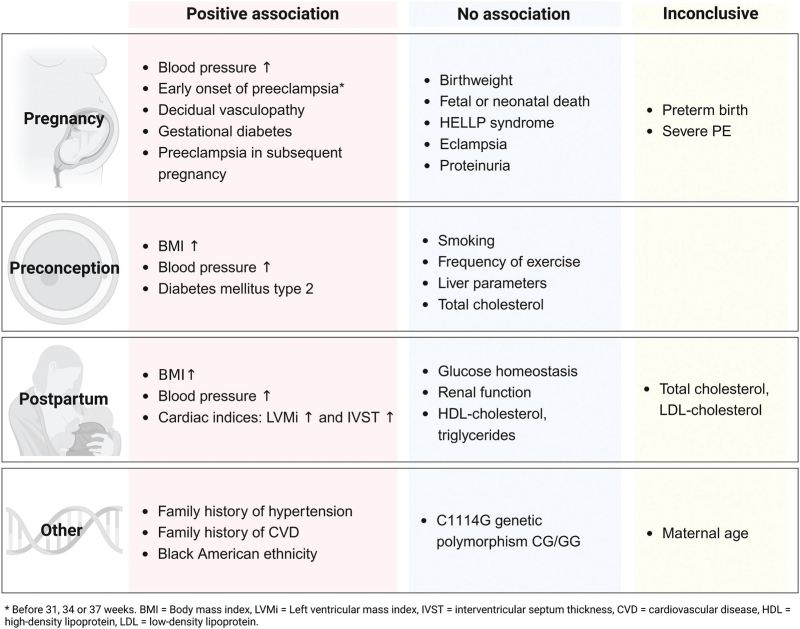
Risk factors and associations with chronic hypertension after preeclampsia.

The characteristics of the included studies are shown in Table 1. Most studies were retrospective cohorts (*N* = 18). Preeclampsia case numbers varied widely, ranging from 20 to 52 691 women. Follow-up duration, reported as mean, median, or range, spanned from 5 to 46 years, with an overall average of 11.1 years across all studies. The diagnosis of preeclampsia was derived from medical records in 19 studies, all of which defined preeclampsia as the presence of hypertension and proteinuria. Six studies used ICD codes to identify women with preeclampsia, and one study relied on self-report. The incidence or prevalence of chronic hypertension was derived from diagnosis codes (in six studies), drug registry data (in one study), medical records (in two studies), or blood pressure measurements at the study follow-up (twelve studies). Five studies used surveys and self-reported chronic hypertension or antihypertensive medication use as the outcome measurements. All studies defined hypertension as SBP at least 140 mmHg or DBP at least 90 mmHg, or use of antihypertensive drugs, except for one study which used a lower cut-off (SBP ≥130 mmHg or DBP ≥80 mmHg, or use of antihypertensive drugs) [16]. We contacted authors via email when crude patient data were not available. Despite our attempts, none of the authors responded to our requests for patient data. The quality scores of the included studies ranged from 5 to 9, with a median score of 8. No studies were rated as poor quality. The NOS quality score evaluation for all included studies is presented in Supplementary Table S1. A total of 30 risk factors were identified. Figure 2 displays all the reported risk factors and effect directions.

**TABLE 1:** Characteristics of included studies

Nr.	First author last name	Year of publication	Study design	Country	Risk factors (*n*)	Preeclampsia cases (*n*)	Follow-up duration (years)	NOS quality score
1	Auger	2017	PCS	Canada	1	39 559	15.5	8
2	Berends	2008	CCS	Netherlands	1	45	7	8
3	Cho	2019	RCS	South Korea	6	1910	8	6
4	Clemmensen	2020	CCS	Denmark	1	49	12	8
5	Engeland	2015	RCS	Norway	2	52.691	46	9
6	Gaugler	2008	CCS	Netherlands	1	20	5.5	8
7	Ghossein-Doha	2013	RCS	Netherlands	13	349	6	6
8	Gronningsaeter	2022	RCS	Norway	1	69	7	8
9	Haßdenteufel	2023	RCS	Germany	1	22 744	4.7	8
10	Haug	2018	RCS	Norway	1	1092	7	8
11	Hooijschuur	2023	RCS	Netherlands	11	185	11	8
12	Kivela	2023	PCS	Finland	11	1357	10	8
13	Kvehaugen	2014	CCS	Norway	1	934	15.1	7
14	Lazdam	2012	RCS	United Kingdom	1	90	9.75	8
15	Lykke	2009	RCS	Denmark	4	33 826	12.9	9
16	Magnussen	2009	PCS	Norway	1	737	16.5	8
17	Marín	2000	CCS	Spain	1	94	13.6	8
18	Portelinha	2009	RCS	Portugal	1	65	6	6
19	Shahbazian	2011	RCS	Iran	1	35	5.7	8
20	Sibai	1991	RCS	United States	1	125	5.4	8
21	Sibai	1992	RCS	United States	1	223	7.2	8
22	Sibai	1986	RCS	United States	3	406	6.6	7
23	Simon	2023	RCS	France	2	37 043	5–10	8
24	Spaan	2012	RCS	Netherlands	9	339	6	8
25	Stevens	2015	RCS	Netherlands	1	119	9.5	5
26	Stuart	2018	RCS	United States	1	3687	26.9	6

CCS, case-control study; PCS, prospective cohort study; RCS, retrospective cohort study.

### Maternal characteristics

Maternal age in relation to chronic hypertension was described in five studies [16–20]. Two studies reported that women who developed chronic hypertension after pregnancy were on average 2–4 years older at the time of conception, or at delivery compared to those who did not develop chronic hypertension [17,19]. In the other three studies, of which two included women from the same hospital population, age at onset of preeclampsia or at postpartum visit was not different in women who did or did not develop chronic hypertension [16,18,20].

One study reported chronic hypertension according to clinician-assigned ethnicity, showing that Black Americans have a higher incidence of chronic hypertension than non-Black Americans after preeclampsia [21].

### Genetic factors

Two studies showed that for women with preeclampsia, having a first-degree family member with hypertension is associated with an increased risk of chronic hypertension, even after correcting for age, smoking, and BMI [17,22]. In addition, having a first-degree family member with other cardiovascular diseases is associated with an increased risk of developing chronic hypertension [22].

No significant association was found between a family history of preeclampsia in mothers and sisters and chronic hypertension 5 years postpregnancy [19].

Moreover, the polymorphism rs4606 in the *Regulator of G protein signaling 2* (*RGS2*) gene was unrelated to the development of chronic hypertension [23].

### Maternal lifestyle factors

One study found no association between prepregnancy smoking or exercise frequency, and the incidence of chronic hypertension after pregnancy [17].

A higher prepregnancy BMI in women with later chronic hypertension was reported in two studies [17,19]. Additionally, the association between maternal postpartum BMI and the incidence of postpartum hypertension was described in three studies with overlapping study populations [16,18,22]. In the largest of these three overlapping studies, including 349 women, a higher BMI at postpartum screening yielded a hazard ratio of 1.11 (1.04–1.18, *P* < 0.01) for the development of hypertension [18].

### Blood pressure

Five studies investigated blood pressure as a risk factor for the development of chronic hypertension [16–19,22]. High prepregnancy blood pressure (SBP 130–139 and DBP 86–89) was found to be associated with chronic hypertension [17].

During pregnancy, higher blood pressure at the first antenatal visit was significantly associated with later chronic hypertension [19].

Ghossein-Doha *et al.* reported that a higher blood pressure, as well as prehypertension (defined as SBP 120–139 mmHg and DBP 80–89 mmHg) at 8 months postpartum, were predictive of chronic hypertension during a mean follow-up of 6 years [18]. This result was confirmed by two other studies conducted at the same hospital [16,22].

### Laboratory parameters

Laboratory parameters for metabolic, hepatic, and renal functions were assessed in four studies [16–18,22]. One study measured total cholesterol (TC), aspartate aminotransferase (AST), alanine aminotransferase (ALT), and gamma-glutamyl transferase (GGT) levels during the preconception period and found no association with chronic hypertension [17]. Glomerular filtration rate, microalbuminuria, triglycerides, glucose, insulin, and homeostatic model insulin resistance (HOMA-IR), assessed in normotensive women during postpartum screening, were also not associated with chronic hypertension [16,18,22]. Spaan *et al.* initially did not find significant differences in cholesterol [total, low-density lipoprotein (LDL), and high-density lipoprotein (HDL)] levels between women with and without chronic hypertension over 5 years after pregnancy, but the same research group later reported higher postpartum LDL and total cholesterol levels in women who later developed hypertension [16]

Finally, Kivela *et al.*[19] reported no significant difference in maximum proteinuria in g/24 h during index pregnancy with preeclampsia between women with and without hypertension.

### Cardiac function

In two studies, extensive cardiac function tests were performed on women who were normotensive at the time of assessment during postpartum screening, occurring an average of 8 months postpartum. Both studies were conducted at the same hospital [16,18]. A higher postpartum left ventricular mass index (LVMi) and interventricular septum thickness (IVST) were positively associated with the development of chronic hypertension.

### Gestational age at diagnosis of preeclampsia

**FIGURE 3:**
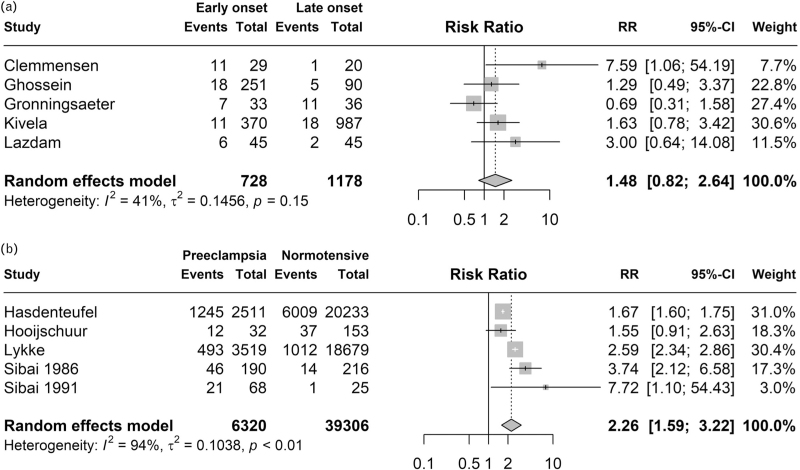
Meta-analyses of risk factors (a) early-onset preeclampsia (<34 weeks gestational age) and (b) preeclampsia in a subsequent pregnancy. Events is having chronic hypertension after a follow-up period of 5 years.

Ten studies described gestational age at the diagnosis of preeclampsia as a potential risk factor for chronic hypertension. Early-onset preeclampsia (eoPE), with onset at less than 34 weeks gestational age, was evaluated in nine studies [9,16,18,19,24–28]. Seven of these studies showed that eoPE resulted in increased risks or incidence of chronic hypertension, one study mentioned that there was no increased risk, and one study observed a nonsignificant lower relative risk of chronic hypertension [26]. Of these studies, the two that partly assessed the same study population showed a positive association [16,18]. When combining individual patient data reported in five studies and performing a meta-analysis, a higher risk ratio for chronic hypertension was found in women with eoPE, although not significant (RR 1.48, 95% CI 0.82–2.64) (Fig. 3a)[18,19,25–27].

In three studies, different cut-offs for gestational age at the onset of preeclampsia were assessed as risk factors for chronic hypertension. Sibai *et al.*[21] classified onset less than 31 weeks gestational age as eoPE and observed an increased risk of developing chronic hypertension in this group versus women aged greater than 31 weeks (OR = 2.3, *P* = 0.01). Two studies compared onset at less than 37 weeks gestational age versus onset at greater than 37 weeks gestational age. The largest of these two studies (*n* = 37 043) observed a higher hazard ratio for chronic hypertension in the more than 37 weeks group after adjusting for several confounders, including maternal age, obesity, smoking, and family history (4.53, 95% CI 3.73–5.51) [9]. No significant association was found in another study (involving 349 women) [18].

In conclusion, an earlier onset of preeclampsia is associated with a higher risk of chronic hypertension more than 5 years after pregnancy.

### Gestational age at birth

Seven studies evaluated gestational age at birth as a potential risk factor [16,18,19,29–32]. Among these, four studies reported an increased risk of chronic hypertension in women who delivered less than 37 weeks gestational age compared to those who delivered more than 37 weeks gestational age [16,29–31]. Conversely, three other studies did not observe an altered risk of chronic hypertension [18,19,32], whereas one study found a significantly lower gestational age at birth in women who developed chronic hypertension, although the calculated hazard ratio was not significantly different from 1 [18]. Hence, it remains inconclusive whether preterm birth, defined as delivery before 37 weeks of gestational age, is significantly associated with an elevated risk of chronic hypertension later in life.

### Preeclampsia, eclampsia, and hemolysis, elevated liver enzymes, and low platelet syndrome

Two studies investigated severe preeclampsia and/or hemolysis, elevated liver enzymes, and low platelet syndrome (HELLP) as a risk factor for chronic hypertension [16,31]. The first study defined severe preeclampsia as having SBP more than 160 mmHg, DPB greater than 110 mmHg, proteinuria greater than 5 g/24 h, or the presence of cerebral disturbances or pulmonary edema, or presence of HELLP syndrome or eclampsia, and found that severe preeclampsia was associated with a significantly increased risk of developing chronic hypertension later in life compared to mild preeclampsia (adjusted hazard ratio [aHR) 3.61, 95% CI 3.43–3.80] [31].

Another study observed no difference in hypertension at follow-up between women with preeclampsia with and without HELLP syndrome [16]. Finally, women with eclampsia tend to exhibit a lower incidence of chronic hypertension (6%) than preeclamptic women without eclampsia (38%), but this was not statistically significant [33]. Additionally, in women with eclampsia, a lower gestational age at diagnosis (<31 weeks) was associated with an increased risk of chronic hypertension compared to those diagnosed at 37–41 weeks gestational age (17.9 vs. 4.8%) [34]. In conclusion, the relationship between severe preeclampsia and chronic hypertension is inconclusive, with studies showing mixed results regarding the association with HELLP and eclampsia.

### Other pregnancy-related outcomes

Birthweight was investigated in five studies, of which three had an overlap in the study population [16,18,19,22,31]. In all of these studies, both birth weight and/or small-for-gestational age, defined as birth weight either below the 10th percentile or 2 SD below the mean, were not associated with the incidence of chronic hypertension.

Fetal and perinatal deaths were not associated with the risk of chronic hypertension in one study [18].

One study observed a higher prevalence of gestational diabetes mellitus (GDM) (31 vs. 12%, *P* = 0.002) and preexisting diabetes type 2 (3.4 vs. 0.3%, *P* = 0.006) before pregnancy in women with preeclampsia who developed chronic hypertension [19]. Lastly, decidual vasculopathy during placental pathological assessment was associated with an increased risk of chronic hypertension in one study [35].

### Subsequent pregnancy with hypertensive disorders

Twelve studies described risk of chronic hypertension after the first pregnancy with preeclampsia based on the presence of preeclampsia or gestational hypertension in a subsequent pregnancy [10,16,18,21,22,30–32,36–39]. Except for one small study (including 20 women) [38], all effect measures reported in these studies point to an increased risk of chronic hypertension when a second pregnancy is also complicated by preeclampsia compared to women with a normotensive subsequent pregnancy.

When performing a meta-analysis including 45 626 women from five studies, we observed a RR of 2.26 (1.59–3.22) for the development of chronic hypertension in women with a second pregnancy complicated by preeclampsia (Fig. 3b) [10,16,21,31,36]. In contrast, Kivela *et al.*[19] reported that for women with preeclampsia included in their study, the risk of chronic hypertension at follow-up was similar between women with and without prior pregnancy with preeclampsia.

## DISCUSSION

### Summary of findings

In this review, we describe the risk factors associated with the development of chronic hypertension in women with preeclampsia 5 years after pregnancy. Our comprehensive search and analysis revealed 32 possible risk factors, of which 14 showed a positive association with increased risk for the development of chronic hypertension: a higher BMI or blood pressure during the preconception or postpartum period, increased LVMi or IVST at postpartum assessment, eoPE (<34 weeks gestational age), preterm preeclampsia (<37 weeks), GDM, presence of decidual vasculopathy, and subsequent pregnancies affected by preeclampsia. No associations were found for preconceptional smoking and exercise, fetal birth weight, perinatal death, HELLP syndrome, eclampsia, liver parameters, and total cholesterol measured before pregnancy, or measures for glucose homeostasis, renal function, and HDL cholesterol or triglyceride postpartum. The literature is inconclusive regarding the association between maternal age, preterm birth, severe preeclampsia, and postpartum measured TC and LDL cholesterol levels with chronic hypertension after pregnancy.

### Interpretation and comparison with existing literature

#### established risk factors for chronic hypertension and cardiovascular disease

Our findings align with the broader literature on established cardiovascular and metabolic risk factors that identifies obesity and type 2 diabetes as significant risk factors for chronic hypertension [40].

Although our study found no association between smoking and lack of physical exercise with chronic hypertension after preeclampsia, the well documented effects of these behaviors on overall cardiovascular health suggest a latent risk that may not surface within the immediate postpartum period but could contribute cumulatively over time [40,41].

Dyslipidemia is traditionally associated with an increased risk of chronic hypertension [40]. Our review yielded inconclusive results regarding LDL and total cholesterol in patients with a history of preeclampsia and risk of later chronic hypertension and no associations for HDL cholesterol and triglycerides.

Additionally, the increased risk of chronic hypertension after preeclampsia observed in individuals with familial predispositions underscores the genetic component of chronic hypertension [42]. In our review, we included one study that investigated a specific genetic polymorphism; however, it found no association with development of chronic hypertension after preeclampsia [23].

As a result, of the established risk factors for cardiovascular disease, maternal BMI, preexisting diabetes, and familial cardiovascular disease showed the greatest potential for inclusion in risk assessment models.

#### Pregnancy-related risk factors

Previous studies have established associations between eoPE and subsequent preeclampsia with the development of hypertension, but these have typically also included follow-up periods as short as 12 months postpartum [8,43]. Our study extends this knowledge by confirming that these risks persist for at least 5 years postpregnancy, highlighting the importance of these risk factors for risk stratification. Our study found no associations between HELLP syndrome or eclampsia and the development of chronic hypertension, suggesting that these conditions may not independently influence long-term hypertensive risk after preeclampsia. Based on the current literature, the association between severe preeclampsia and later hypertension remains inconclusive, and we speculate that this might be due to the lack of a standardized definition and use in clinical practice.

Our results further indicate that GDM is a risk factor for chronic hypertension, a relationship that holds even in pregnant women without preeclampsia [44]. Although this might be attributed to shared risk factors already present before pregnancy, causal pathways might include insulin resistance, which exacerbates GDM, leading to elevated blood pressure by promoting vascular smooth muscle proliferation and sympathetic nervous system activity [45]. Moreover, GDM may trigger inflammatory responses and endothelial dysfunction, further contributing to hypertension by increasing systemic vascular resistance and impairing vascular tone [45].

Additionally, decidual vasculopathy in placental histopathological assessment is associated with chronic hypertension. The lesions in decidual vasculopathy, also known as acute atherosis, are characterized by fibrinoid deposits, thickened vessel walls, narrowed lumens, and inflammatory cell infiltration, and resemble those in atherosclerosis, potentially explaining the underlying mechanism of this association [46]. If a pathological assessment is conducted, identifying this condition could provide significant additional value for assessing the risk of chronic hypertension.

Our study identified no significant associations between fetal outcomes, including birth weight, fetal or neonatal death, and the development of chronic hypertension after preeclampsia. The relationship between preterm birth and chronic hypertension remains inconclusive, and to our knowledge, this aspect has not been extensively reviewed.

#### Physiological and biochemical markers

Our systematic review identified a consistent association across multiple studies between elevated blood pressure measurements at various stages, preconceptionally, at the first prenatal visit, during an eclamptic insult, and 8 months postpartum, and an increased risk of chronic hypertension more than 5 years postpregnancy. This association likely reflects a preexisting trend of rising blood pressure preceding the development of hypertension.

Moreover, an increased and increased IVST on echocardiography performed in normotensive women during postpartum screening at 8 months postpartum are associated with the development of chronic hypertension. The observed increases in the LVMi and IVST could be a residual effect of hypertension experienced during preeclampsia, despite a return to normotensive levels postpartum. These cardiac measurements, which are also typically elevated in individuals with existing hypertension [47], suggest their potential utility as risk stratification tools for postpartum women.

In the reviewed studies, laboratory parameters relating to metabolic, hepatic, and renal functions measured before or after pregnancy generally showed no association with chronic hypertension more than 5 years after pregnancy. Consequently, incorporating laboratory parameters into risk stratification models for predicting long-term chronic hypertension postpregnancy does not seem to offer a significant utility. Notably, our search did not identify studies exploring the association between angiogenic markers, such as soluble fms-like tyrosine-kinase 1 (sFlt-1) and placental growth factor (PlGF), with chronic hypertension 5 years after pregnancy, although one study showed no association with chronic hypertension 1 year after pregnancy [48]. Lastly, aspirin use was reported in only one study, in which the association with the development of chronic hypertension has not been investigated [35]. This lack of information can be due to the fact that aspirin use is only recently part of common clinical practice, with many included studies published several years ago or using data before introduction of aspirin as a preventive medicine.

### Strengths and limitations

Our systematic review makes a unique contribution by identifying all known risk factors for chronic hypertension following preeclampsia, a gap not explicitly addressed in previous reviews. Unlike prior studies, such as Groenhof *et al.*[49] in 2017 and Hutchesson *et al.*[50] in 2022, we take a broader and more comprehensive approach to risk factors. This overview is essential for developing targeted screening programs and risk models.

Moreover, by calculating relative risks and conducting a meta-analysis, we enhanced the clinical applicability of the data.

However, the meta-analysis revealed substantial heterogeneity (*I*^2^ = 92 and 94% for subsequent pregnancy), which primarily stemmed from differences in incidence of chronic hypertension after preeclampsia between the included studies. This might be explained by differences in study populations, such as specialized hospital cohorts versus population-based cohorts in high-income countries. Meta-regression analyses suggested that older studies reported a higher relative risk for chronic hypertension, which may reflect changes in diagnostic criteria or management practices over time. Despite these variations, excluding studies published before 2000 did not significantly change our overall findings. Furthermore, visual inspection of funnel plots (data not shown) did not indicate significant publication bias. However, it is important to note that the number of studies included in the meta-analysis is quite low, which may limit the ability to detect publication bias.

Other acknowledged limitations of this review arise from the quality of the included studies, particularly owing to their retrospective design and insufficient adjustment for confounders, such as maternal age or BMI [25,37]. Additionally, our review was unable to perform subgroup analyses for women with preexisting type I chronic hypertension (prenatal SBP 130–139 mmHg or DBP 80–89 mmHg) due to insufficient data in the included studies. Women in this subgroup may exhibit distinct risk factors for chronic hypertension later in life, warranting further investigation. Despite these limitations, this review successfully identified several promising risk factors for chronic hypertension 5 years after preeclampsia.

### Future perspectives and clinical application

An increased risk of cardiovascular disease, including heart failure, stroke, and coronary artery disease, has been reported in women with a history of preeclampsia [39,51] and therefore, preeclampsia has been added as a risk factor for cardiovascular disease by the American Heart Association [52]. In addition to being a risk factor, the diagnosis of preeclampsia may also be regarded as an early warning sign and an opportunity to address other risk factors for cardiovascular disease, including chronic hypertension. The development of a screening tool for calculating chronic hypertension risk for use in postpartum surveillance could improve follow-up care after preeclampsia, promote better cardiovascular disease prevention, and contribute to a healthier population. A meaningful screening tool should go beyond an exhaustive list of potential risk factors. We suggest selecting factors with substantial evidence or those that can be easily and affordably implemented in healthcare settings. Based on this systematic review, the strongest evidence suggests that preeclampsia in subsequent pregnancies is a risk factor for chronic hypertension. However, the use of this risk factor during postpartum visits is not possible. Therefore, we propose to use maternal anthropometrics related to cardiovascular disease, such as BMI and maternal blood pressure, in combination with onset of preeclampsia at less than 34 weeks gestational age, as these have shown consistent associations with chronic hypertension after preeclampsia and are easily available in clinical practice.

This approach is further supported by the results of a prospective cohort study, which found that blood pressure and BMI accounted for up to 77% of the excess risk of cardiovascular disease in women with a history of hypertensive disorders of pregnancy, such as preeclampsia [53].

In a more extensive risk assessment, the results of placental pathologic investigation (specifically, the presence of decidual vasculopathy) and cardiac function parameters (e.g. LVMI) could provide additional insights. Naturally, a new integrated risk score should be prospectively investigated in the clinical setting before implementation.

In conclusion, although current practice has become aware of the increased risk of cardiovascular disease after preeclampsia, and there is more and more attention for monitoring cardiovascular health after preeclampsia, we have yet to identify patients that could benefit most from the developed surveillance programs. In this systematic review, we identified 14 different risk factors that affect the risk of developing chronic hypertension 5 years after preeclampsia. eoPE and preeclampsia in more than one pregnancy seem to be the most important risk factors for clinical practice.

## ACKNOWLEDGEMENTS

We thank Dr M.F.M. Engel, a biomedical information specialist from the Medical Library of Erasmus University Medical Center, for assisting us with the literature search.

We would like to acknowledge Dr Sten P. Willemsen, biostatistician at Erasmus Medical Center, for his consultation on the statistical analysis of our meta-analysis.

Preliminary data was presented as a poster at the 71st Annual Meeting of the Society for Reproductive Investigation (SRI), 12–16 March 2024, in Vancouver.

Results were also presented in a short oral presentation at the 33rd European Meeting on Hypertension and Cardiovascular Renal Protection (ESH 2024), Berlin, from 31 May to 3 June 2024.

Funding: no funding was received for this research.

### Conflicts of interest

There are no conflicts of interest.

## Supplementary Material

Supplemental Digital Content
