# Supplementary material for: Risk factors for chronic hypertension 5 years after a pregnancy complicated by preeclampsia: a systematic review and meta-analysis
Source: J Hypertens. 2025 Feb 27;43(6):939–48. doi: 10.1097/HJH.0000000000003995 (PMC12052050; doi:10.1097/HJH.0000000000003995)
Supplement: Supplemental Digital Content [file jhype-43-0939-s001.docx]

**Supplementary material**

**Supplementary table 1: Quality assessment Newcastle-Ottowa Scale**

| Nr. | First Author | Year of publication | Selection (max 4) | Comparability (max 2) | Outcome (max 3) | Total score (max 9) | Quality Rating (Good, Fair, Poor) |
| --- | --- | --- | --- | --- | --- | --- | --- |
| 1 | Auger | 2017 | *** | ** | *** | 8 | Good |
| 2 | Berends | 2008 | **** | * | *** | 8 | Good |
| 3 | Cho | 2019 | **** | * | *** | 7 | Good |
| 4 | Clemmensen | 2020 | **** | * | *** | 8 | Good |
| 5 | Engeland | 2015 | **** | ** | *** | 9 | Good |
| 6 | Gaugler | 2008 | **** | * | *** | 8 | Good |
| 7 | Ghossein-Doha | 2013 | **** | * | ** | 7 | Good |
| 8 | Gronningsaeter | 2022 | **** | * | *** | 8 | Good |
| 9 | Haßdenteufel | 2023 | **** | * | *** | 8 | Good |
| 10 | Haug | 2018 | *** | ** | *** | 8 | Good |
| 11 | Hooijschuur | 2023 | **** | * | *** | 8 | Good |
| 12 | Kivela | 2023 | **** | * | *** | 8 | Good |
| 13 | Kvehaugen | 2014 | *** | ** | ** | 7 | Good |
| 14 | Lazdam | 2012 | **** | * | *** | 8 | Good |
| 15 | Lykke | 2009 | **** | ** | *** | 9 | Good |
| 16 | Magnussen | 2009 | *** | ** | *** | 8 | Good |
| 17 | Marín | 2000 | **** | * | *** | 8 | Good |
| 18 | Portelinha | 2009 | **** | * | * | 6 | Fair |
| 19 | Shahbazian | 2011 | **** | * | *** | 8 | Good |
| 20 | Sibai | 1986 | **** | * | *** | 8 | Good |
| 21 | Sibai | 1991 | **** | * | *** | 8 | Good |
| 22 | Sibai | 1992 | *** | * | *** | 7 | Good |
| 23 | Simon | 2023 | **** | * | *** | 8 | Good |
| 24 | Spaan | 2012 | **** | ** | ** | 8 | Good |
| 25 | Stevens | 2015 | *** | * | * | 5 | Fair |
| 26 | Stuart | 2018 | ** | ** | ** | 6 | Fair |

**Appendix 1: Search strategy**

| **Database searched** | **Platform** | **Years of coverage** | **Records** | **Records after duplicates removed** |
| --- | --- | --- | --- | --- |
| Medline ALL | Ovid | 1946 - Present | 3705 | 3686 |
| Embase | Embase.com | 1971 - Present | 5546 | 2438 |
| Web of Science Core Collection* | Web of Knowledge | 1975 - Present | 3206 | 395 |
| Cochrane Central Register of Controlled Trials | Wiley | 1992 - Present | 368 | 223 |
| Additional Search Engines: Google Scholar*** (200 top-ranked) | | | 200 | 55 |
| **Total** | | | **13025** | **6797** |

*Science Citation Index Expanded (1975-present) ; Social Sciences Citation Index (1975-present) ; Arts & Humanities Citation Index (1975-present) ; Conference Proceedings Citation Index–Science (1990-present) ; Conference Proceedings Citation Index–Social Science & Humanities (1990-present) ; Emerging Sources Citation Index (2005-present)

***Google Scholar was searched via "Publish or Perish" to download the results in EndNote.

No other database limits were used than those specified in the search strategies

exclude conference abstracts from 2020 and older

exclude case reports

**Embase 5546**

('eclampsia and preeclampsia'/exp OR 'HELLP syndrome'/de OR (eclamp* OR preeclamp* OR pre-eclamp* OR preclamp* OR ((toxemia*) NEAR/3 (pregnan*)) OR hellp):ab,ti,kw) **AND** (((puerperium/de OR 'longitudinal study'/de OR 'follow up'/de) AND (hypertension/de OR 'elevated blood pressure'/de OR 'antihypertensive agent'/exp OR 'cardiovascular risk'/de)) OR (((postpart* OR post-part* OR puerperium* OR puerperal* OR chronic OR persist* OR 1-year* OR 2-year* OR 3-year OR 4-year* OR 5-year* OR 12-month* OR 24-month* OR 36-month* OR 48-month* OR 60-month* OR longitudinal OR long-term* OR longterm OR longer OR sustain* OR after-preeclamp* OR post-preeclamp* OR history-of-preeclam* OR formerly-preeclam* OR subsequent* OR late* OR year* OR follow-up* OR followup*) NEAR/3 (hypertens* OR high-blood-press* OR elevat*-blood-press* OR increas*-blood-press* OR antihypertens* OR anti-hypertens* OR cardiovascular*))):ab,ti,kw) NOT (([Conference Abstract]/lim OR [Conference Review]/lim) AND [1800-2020]/py) NOT ('case report'/de OR (case-report):ti)

**Medline**

(exp Hypertension, Pregnancy-Induced/ OR (eclamp* OR preeclamp* OR pre-eclamp* OR preclamp* OR ((toxemia*) ADJ3 (pregnan*)) OR hellp).ab,ti,kf.) **AND** (((Postpartum Period/ OR Longitudinal Studies/ OR Follow-Up Studies/) AND (Hypertension/ OR exp Antihypertensive Agents/ OR exp Heart Disease Risk Factors/)) OR (((postpart* OR post-part* OR puerperium* OR puerperal* OR chronic OR persist* OR 1-year* OR 2-year* OR 3-year OR 4-year* OR 5-year* OR 12-month* OR 24-month* OR 36-month* OR 48-month* OR 60-month* OR longitudinal OR long-term* OR longterm OR longer OR sustain* OR after-preeclamp* OR post-preeclamp* OR history-of-preeclam* OR formerly-preeclam* OR subsequent* OR late* OR year* OR follow-up* OR followup*) ADJ3 (hypertens* OR high-blood-press* OR elevat*-blood-press* OR increas*-blood-press* OR antihypertens* OR anti-hypertens* OR cardiovascular*))).ab,ti,kf.) NOT ((news OR congres* OR abstract* OR book* OR chapter* OR dissertation abstract*).pt. AND 1800:2020.(sa_year).) NOT (Case Reports/ OR (case-report).ti.)

**Cochrane**

((eclamp* OR preeclamp* OR pre NEXT eclamp* OR preclamp* OR ((toxemia*) NEAR/3 (pregnan*)) OR hellp):ab,ti) **AND** ((((postpart* OR post NEXT part* OR puerperium* OR puerperal* OR chronic OR persist* OR 1 NEXT year* OR 2 NEXT year* OR 3 NEXT year OR 4 NEXT year* OR 5 NEXT year* OR 12 NEXT month* OR 24 NEXT month* OR 36 NEXT month* OR 48 NEXT month* OR 60 NEXT month* OR longitudinal OR long NEXT term* OR longterm OR longer OR sustain* OR after NEXT preeclamp* OR post NEXT preeclamp* OR history NEXT of NEXT preeclam* OR formerly NEXT preeclam* OR subsequent* OR late* OR year* OR follow-up* OR followup*) NEAR/3 (hypertens* OR high NEXT blood NEXT press* OR elevated NEXT blood NEXT press* OR increased NEXT blood NEXT press* OR antihypertens* OR anti-hypertens* OR cardiovascular*))):ab,ti) NOT "conference abstract":kw

**Web of Science**

TS=(((eclamp* OR preeclamp* OR pre-eclamp* OR preclamp* OR ((toxemia*) NEAR/2 (pregnan*)) OR hellp)) **AND** ((((postpart* OR post-part* OR puerperium* OR puerperal* OR chronic OR persist* OR 1-year* OR 2-year* OR 3-year OR 4-year* OR 5-year* OR 12-month* OR 24-month* OR 36-month* OR 48-month* OR 60-month* OR longitudinal OR long-term* OR longterm OR longer OR sustain* OR after-preeclamp* OR post-preeclamp* OR history-of-preeclam* OR formerly-preeclam* OR subsequent* OR late* OR year* OR follow-up* OR followup*) NEAR/2 (hypertens* OR high-blood-press* OR elevated-blood-press* OR increased-blood-press* OR antihypertens* OR anti-hypertens* OR cardiovascular*))))) NOT (DT=(Meeting Abstract OR Meeting Summary) AND py=(1800-2020)) NOT TI=(case-report)

**Google Scholar**

eclampsia|preeclampsia "postpartum|puerperium|puerperal|persistent|sustained|chronic|subsequent hypertension|hypertensive|antihypertensives"

eclampsia|preeclampsia 'postpartum|puerperium|puerperal|persistent|sustained|chronic|subsequent hypertension|hypertensive|antihypertensives'
